# Global prediction model for COVID-19 pandemic with the characteristics of the multiple peaks and local fluctuations

**DOI:** 10.1186/s12874-022-01604-x

**Published:** 2022-05-13

**Authors:** Haoran Dai, Wen Cao, Xiaochong Tong, Yunxing Yao, Feilin Peng, Jingwen Zhu, Yuzhen Tian

**Affiliations:** 1 School of Geoscience and Technology, Zhengzhou University, Zhengzhou, 450001 China; 2 School of Geospatial Information, University of Information Engineering, Zhengzhou, 450001 China

**Keywords:** COVID-19, Epidemic prediction, Logistic growth function, NO_2_ concentrations, Compartmental model

## Abstract

**Background:**

With the spread of COVID-19, the time-series prediction of COVID-19 has become a research hotspot. Unlike previous epidemics, COVID-19 has a new pattern of long-time series, large fluctuations, and multiple peaks. Traditional dynamical models are limited to curves with short-time series, single peak, smoothness, and symmetry. Secondly, most of these models have unknown parameters, which bring greater ambiguity and uncertainty. There are still major shortcomings in the integration of multiple factors, such as human interventions, environmental factors, and transmission mechanisms.

**Methods:**

A dynamical model with only infected humans and removed humans was established. Then the process of COVID-19 spread was segmented using a local smoother. The change of infection rate at different stages was quantified using the continuous and periodic Logistic growth function to quantitatively describe the comprehensive effects of natural and human factors. Then, a non-linear variable and NO_2_ concentrations were introduced to qualify the number of people who have been prevented from infection through human interventions.

**Results:**

The experiments and analysis showed the *R*^2^ of fitting for the US, UK, India, Brazil, Russia, and Germany was 0.841, 0.977, 0.974, 0.659, 0.992, and 0.753, respectively. The prediction accuracy of the US, UK, India, Brazil, Russia, and Germany in October was 0.331, 0.127, 0.112, 0.376, 0.043, and 0.445, respectively.

**Conclusion:**

The model can not only better describe the effects of human interventions but also better simulate the temporal evolution of COVID-19 with local fluctuations and multiple peaks, which can provide valuable assistant decision-making information.

**Supplementary Information:**

The online version contains supplementary material available at 10.1186/s12874-022-01604-x.

## Background

**Fig. 1:**
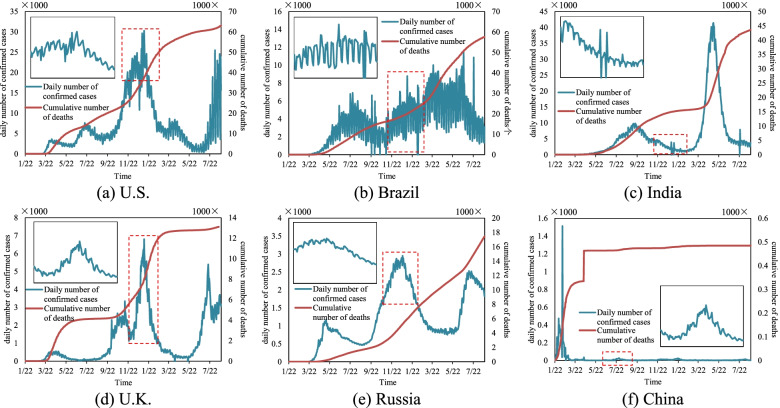
The epidemic Curves of COVID-19 in the word

The rapid spread of COVID-19 brought unprecedented harm to human life, economic development, and social stability. How to control the spread of COVID-19 in a way that minimizes the risk and the cost has become the focus of research. A timely grasp of the characteristics of spread and future development of COVID-19 can turn passive prevention and control into the initiative. However, the prevalence of COVID-19 is influenced by a combination of subjective factors (such as population activity and human control) and objective factors (such as temperature, humidity, and social economy) [1–3], which leads to a new curve form of long-time series, large fluctuations, and multiple peaks. As shown in Fig. 1, the epidemic duration is long (more than 2 years), and the trends of the rising and falling curves are asymmetrical and not smooth.

The researches on the prediction of COVID-19 can be mainly divided into three parts: classical dynamical models of infectious diseases, time-series prediction models, and multivariate prediction models. The time-series prediction models [4–6] use time-series forecasting methods to solve the problem of prediction, such as long short-term memory (LSTM), sliding window averaging, and Autoregressive Integrated Moving Average model (ARIMA). However, these models only mine the laws of changes in the time series of the epidemic curves and still lack the consideration of influencing factors, only for short-term predictions. The multivariate prediction models [7–9] use regression methods to establish the relationship between the number of confirmed cases and correlated factors. However, the effects of these factors are not combined with the transmission chain and cannot explain how they affect the spread of COVID-19. The dynamical models of infectious diseases divide the population into different groups based on the epidemiological characteristics of individuals and use differential equations to express the process of contact infection between populations. It has two advantages over the above two approaches: it can represent the dynamical process of infectious diseases (susceptible - infected - recovered); its epidemiological parameters are important for the prevention and control of epidemics and pathological studies. Therefore, dynamical models are gradually becoming the mainstream mathematical approaches for the researches of infectious diseases. For example, SEIQR [10], SIR-X [11], and SIQR [12], SEIRD [13], SEIRS [14], e-ISHR [15], exponential and non-linear growth model [16] and new infectious disease models by adding asymptomatic infectors [17–19] and environmental infection [20, 21]. However, they are usually only suitable for single-peaked and short-term prediction, and lack consideration of multiple human or natural factors leading to the predicted curves presenting a smooth and symmetric form.

Huang firstly proposed the Global Prediction System for COVID-19 Pandemic (GPCP) by combining dynamical models with meteorological factors [8]. Among them, the daily number of confirmed cases (*dI*) was quantified by introducing infection rate (*β*) and adjustment parameters (*ε*), i.e., *dI* = *βI*-*εI*^2^. The item (*εI*^2^) expresses the number of infected people reduced due to human interventions. But when the number of confirmed cases (*I*) is large, the value of this item (*εI*^2^) is much greater than the number of new infections per day (*βI*), which will lead to the daily number of confirmed cases (*dI*) being negative numbers. Therefore, this method is more suitable for early short epidemic prediction (*I* is relatively small). Besides, this system did not predict epidemics with multiple peaks. Subsequently, a second-generation global prediction system was proposed [22] and simulated the second wave of the outbreak. Because the dynamical models are a forward flow model, the number of susceptible individuals keeps decreasing and there will be no one infected at the end, which makes it impossible for the infection curve to continue to rise. Thus, these methods are not suitable to describe the characteristics of the development of a prolonged outbreak.

In summary, the current dynamical prediction models have the following shortcomings:①most models are only for smooth and symmetric curves with the short time and single peak; ②most models lack integrated consideration of human prevention and control, environmental factors, and transmission mechanisms of infectious disease; ③a large amount of unknown data is included in the models, which will lead to difficulty in verifying the results and bring more ambiguity and uncertainty. This study proposed novel prediction models for COVID-19, aiming to better describe epidemic curves with the long time, multiple peaks, and high fluctuations and provide valuable auxiliary decision-making information. Firstly, considering the virus mutations and the effectiveness of the vaccine, the circular SEAICR_loop_ model was proposed based on the SEIR model, then the IR_loop_ model was established only by retaining infected humans and removed humans. Secondly, the logistic growth function was used to describe the change laws of infection rate caused by natural factors and human interventions in each stage. Finally, anomalous values of NO_2_ concentrations and nonlinear function were introduced to quantify the number of infected people reduced due to human interventions, which solved the problem of local and large fluctuations in the epidemic curve. The main contributions of this study are as follows:

1. A theoretical prediction model was proposed to describe the epidemic curve with the characteristics of long time series, multi peaks, asymmetry, and local fluctuations. The model is simple and retains the epidemiological significance of model parameters. The parameters can be completely verified by the actual data, which greatly reduces the uncertainty and fuzziness of results.
2. The model uses the logistic growth function to describe the change of infection rate in different stages, which can measure the impact of natural and human factors. At the same time, the NO_2_ concentrations were introduced to quantify the number of infected people reduced due to human interventions, which effectively integrates the characteristics of local fluctuations for epidemic curves.

## Methods

The spread of COVID-19 is influenced synthetically by both natural and human factors. To ensure the accuracy and scientificity of the time-series prediction model, it is necessary to integrate the influence of these factors. Therefore, the SEAICR_loop_ and the IR_loop_ model were proposed based on the SEIR model, and the Logistic function was used to quantify the infection rate under the influences of multiple factors. Meanwhile, the impacts on human intervention were modeled using NO_2_ concentrations outliers.

### Infectious disease model based on characteristics of long-time series

#### SEAICR_loop_ model

The classical dynamical models translate the problem of the change in the number of infected people into mathematical differential equations. Among them, the SEIR and SIR model is the most classic. However, the premise for the use of dynamic models is that population movements in and out are not taken into account. In addition, the dynamical models are a positive one-way population transformation and usually do not return from removed humans (*R*) to susceptible humans (*S*), because the model assumes that people who die or acquire antibodies will not be infected again. However, this is the opposite of COVID-19 infection, and the traditional dynamical models are inoperative for COVID-19 with the characteristics of long-time series and multiple peaks.

**Fig. 2:**
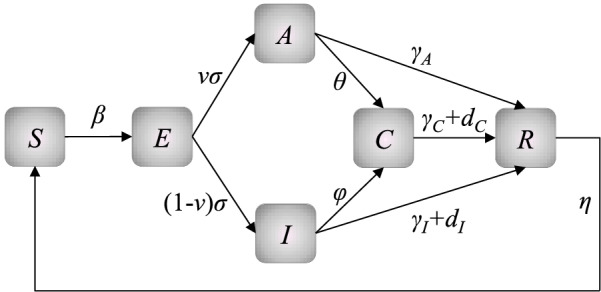
Dynamics diagram of SEAICR_loop_ model

The SEAICR_loop_ model was proposed by improving the classic model, which has a more complete dynamical mechanism. It divides the population into susceptible (*S*), exposed (*E*), asymptomatic (*A*), infected (*I*), detected (*C*), and removed (*R*) humans. At the beginning of the outbreak, all but one or a few migratory infected persons are susceptible (*S*). When they contact effectively with infected cases, they, called the exposed humans (*E*), do not show symptoms immediately. After a period of incubation, a part of exposed humans show clinical symptoms and then become symptomatic infected humans (*I*) and the rest of them still show no symptoms but are infectious, and are called asymptomatic infected humans (*A*). Subsequently, there are two ways for infected humans (*A* and *I*) to exit the transmission system. One way is isolation through testing and they are called the detected infected humans (*C*). The other way is through immunization, treatment, and death and they are called removed humans (*R*). Finally, people who recover will be infected again due to the effectiveness of vaccines and viral mutations, which can achieve a closed-loop transmission mechanism of infectious diseases to adapt to the COVID-19 pandemic. As shown in the following Eqs. (1, 2, 3, 4, 5, 6) and Fig. 2.

1 $${S}_t={S}_1+\sum \limits_{j=1}^{t-1}\left(\eta {R}_j-\beta {S}_j\right)$$

2 $${E}_t={E}_1+\sum \limits_{j=1}^{t-1}\left(\beta {S}_j-\sigma {E}_j\right)$$

3 $${A}_t={A}_1+\sum \limits_{j=1}^{t-1}\left(v\sigma {E}_j-\left(\theta +{\gamma}_A\right){A}_j\right)$$

4 $${I}_t={I}_1+\sum \limits_{j=1}^{t-1}\left(\left(1-v \right)\sigma {E}_j-\left(\varphi +{\gamma}_I+{d}_I\right){I}_j\right)$$

5 $${C}_t={C}_1+\sum \limits_{j=1}^{t-1}\left(\theta {A}_j+\varphi {I}_j-\left({\gamma}_C+{d}_C\right){C}_j\right)$$

6 $${R}_t={R}_1+\sum \limits_{j=1}^{t-1}\left({\gamma}_C{C}_j+{\gamma}_A{A}_j+{\gamma}_I{I}_j-\eta {R}_j\right)$$

Where *β* is the effective transmission rate; *σ* is the progression rate from exposed state to infectious state; *γ* is the recovery rate; *d* is the mortality rate; *θ* and *φ* are the detection rate of asymptomatic and symptomatic infected cases, respectively; *ν* is the fraction of new infectious humans that are asymptomatic; *η* is the proportion of recovered humans who are likely to be infected again.

#### IR_loop_ model

There are a lot of unknowns in the epidemiology of infectious diseases, especially in the face of the sudden outbreak of new infectious diseases, such as the number of exposed humans, the number of asymptomatic infected humans, and the infectivity of the latent period. The actual reported data are only the number of confirmed people, dead people, and recovered people. However, the number of latent people is not known. In this case, it is inaccurate that the dynamical model parameters are estimated using only the numbers of confirmed people, dead people, and recovered people as validation data, which will bring some ambiguity and uncertainty to the prediction results. Okuonghae [17], Alberti [23] and Cao [24] also pointed out that there is great uncertainty in using early sample data to predict the unknown parameters. Therefore, the population is only divided into infected humans and removed humans. Removed humans contain the dead people and recovered people. As shown in the following Eqs. (7 and 8). While the model is simple, it retains the dynamic mechanism of infectious diseases and the significance of epidemiological parameters of model parameters.

7 $${I}_t={I}_1+\sum \limits_{j=1}^{t-1}\left(\beta {I}_j-\gamma {I}_j\right)$$

8 $${R}_t={R}_1+\sum \limits_{j=1}^{t-1}\gamma {I}_j$$

### Improvement of dynamical model parameters under the influences of multiple factors

Classical dynamical models are the ideal transmission of infectious diseases, and their predicted outcomes usually present smooth and standard normal curves. However, the spread of infectious diseases is influenced by a variety of factors, and the infection curve is irregular and has large fluctuations, asymmetry, and multiple peaks. Therefore, dynamical models need to consider the influence of multiple factors.

#### Model of infection rate based on the periodic logistic function

**Fig. 3:**
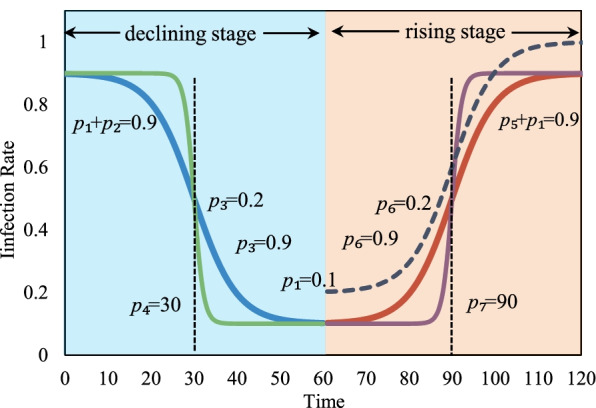
Diagram of Logistic Growth Function (The parameters in the figure are all examples)

Although many factors have different effects on infectious diseases, they can be attributed to the change in infection rate in the dynamic models. For example, human interventions are to reduce the infection rate, and the infection rate of the influenza virus shows seasonal characteristics. In the classical dynamical model, *β* is considered as a constant, which can only be applied when the infectious disease is in the ideal spreading state. At the beginning of an outbreak, COVID-19 is in a state of free transmission and the infection rate is relatively high. As the number of infected people continues to increase, the interventions will start to perform; the infection rate will continue to decrease after some time. When the intensity of interventions is alleviated, COVID-19 may spread again and the infection rate will continue to increase again. This process is very similar to the Logistic growth function in mathematics, as shown in Fig. 3, and its form is shown in Eq. 9 below. The parameters of the Logistic function are estimated by using genetic arithmetic for approximate parameters solution from the corresponding epidemic data.

9 $$\beta =\left\{\begin{array}{l}\kern0.5em {p}_1+\frac{p_2}{1+\exp \left(1+{p}_3\ast \left({p}_4-t\right)\right)},\kern0.5em \mathrm{declining}\ \mathrm{stage}\\ {}\kern0.5em {p}_1+\frac{p_5}{1+\exp \left(1+{p}_6\ast \left(t-{p}_7\right)\right)},\kern0.5em \mathrm{rising}\ \mathrm{stage}\end{array}\right.$$

Where *t* is days. During a declining period of infection rates, *p*_1_ + *p*_2_ is the initial infection rate; *p*_1_ is the eventual infection rate after human prevention and control; *p*_3_ is the hysteresis of human interventions, and the larger its value indicates that the intensity of human interventions is high and the infection rate decreases rapidly. On the contrary, the smaller its value, the intensity of human interventions is low and the infection rate decreases slowly. During a rising period of infection rates, *p*_1_ + *p*_5_ is the eventual infection rate after relaxing the prevention and control; *p*_6_ is the hysteresis of relaxation of human interventions, and the larger its value indicates that the faster the interventions are relaxed, the faster the infection rate will rise. On the contrary, it indicates that the human interventions are relaxed slowly and the infection rate rises slowly. *p*_4_ and *p*_6_ are the inflection point of changes in infection rate. When the virus mutates, the *p*_1_ will not be the same in both periods because it does not belong to the same nature of the virus, and the curve of the rising period changes as the dark purple dashed line in Fig. 3.

#### Model of non-pharmaceutical interventions based on NO_2_ concentration

Dynamical models of infectious diseases usually are smooth curves. However, the actual curve with large fluctuations may be due to the inaccuracy of the human detection on the one hand, and the human interventions on the other hand. Researchers [25–27] worldwide observed reductions in NO_2_ concentrations due to lockdown and related diminished human activities, notably the reduced industrial and vehicular use. In addition, there are also many studies [28, 29] show that a strong correlation between changes in NO_2_ concentrations and COVID-19. NO_2_ concentrations, as the exhaust gases of vehicle emissions and industrial production, can reflect indirectly the human interventions to restrict the work, travel, and activities of people [8, 22]. The impact of human interventions is mainly reflected in the reduction of the number of infected people. Therefore, the parameter *ε* is introduced to express the proportion of the reduction and added to the dynamics model of infectious diseases. The specific improvements are divided into the improvement of the SEAICR_loop_ model and IR_loop_ model, as the following eqs. (11, 12, 13 and 14). The parameter *ε* is linearized by the difference between the NO_2_ concentrations and the concentrations without the human interventions, as in Eq. 10.

10 $$\varepsilon ={\varepsilon}_0+{\varepsilon}_1\left(\overline{C}-C\right)$$

• SEAICR_loop_ Model

11 $${S}_t={S}_1+\sum \limits_{j=1}^{t-1}\left(\eta {R}_j+\varepsilon {I}_j+\varepsilon {A}_j-\beta {S}_j\right)$$

12 $${A}_t={A}_1+\sum \limits_{j=1}^{t-1}\left(v \sigma {E}_j-\left(\theta +{\gamma}_A\right){A}_j-\varepsilon {A}_j\right)$$

13 $${I}_t={I}_1+\sum \limits_{j=1}^{t-1}\left(\left(1-v \right)\sigma {E}_j-\left(\varphi +{\gamma}_I+{d}_I\right){I}_j-\varepsilon {I}_j\right)$$

• IR_loop_ Model

14 $${I}_t={I}_1+\sum \limits_{j=1}^{t-1}\left(\beta {I}_j-\gamma {I}_j-\varepsilon {I}_j\right)$$

where *ε* is the moderating parameter of the epidemic curve, which mainly corresponds to the uninfected people protected due to human prevention and control. $\overline{C}$ is the average NO_2 _concentration without human interventions, and *C* is the daily NO_2_ concentration in μg/*m*^3^.

## Results

To validate the correctness and rationality of the improved model, global epidemic data were collected from the COVID-19 Data Repository by the Center for Systems Science and Engineering (CSSE) at Johns Hopkins [30]. The data period is from January 22, 2020 to November 30, 2021. Among them, the data from January 22, 2020 to September 30, 2021, will be used as the historical epidemic fitting, and the data from October 1, 2021 to November 30, 2021, will be used to verify the future prediction results. Global climate and air quality data were collected from a dedicated dataset provided by the Air Quality Open Data Platform Worldwide COVID-19 dataset (WAQI Project, https://aqicn.org/data-platform/covid19/cn/). The values of relevant parameters are shown in Table 1.

**Table 1:** The values of relevant parameters of dynamical models

Parameter	^*^Baseline value	^*^Range
*p*_1_ ~ *p*_7_	Fitted	Estimated
^*α*^	0.5	[0, 1]
^*υ*^	0.5	[0, 1]
^*σ*^	1/5.2	[1/14, 1/3]
^*φ*^	Fitted	Estimated
^*θ*^	Fitted	Estimated
*γ*_*C*_	1/15	[1/30, 1/3]
*γ*_*I*_, *γ*_*A*_	0.13978	[1/30, 1/3]
*d*_*C*_, *d*_*I*_	0.015	[0.001, 0.1]

^*^Reference from Okuonghae [17] and Cao [24]

### Time-series fitting of historical epidemic data

**Fig. 4:**
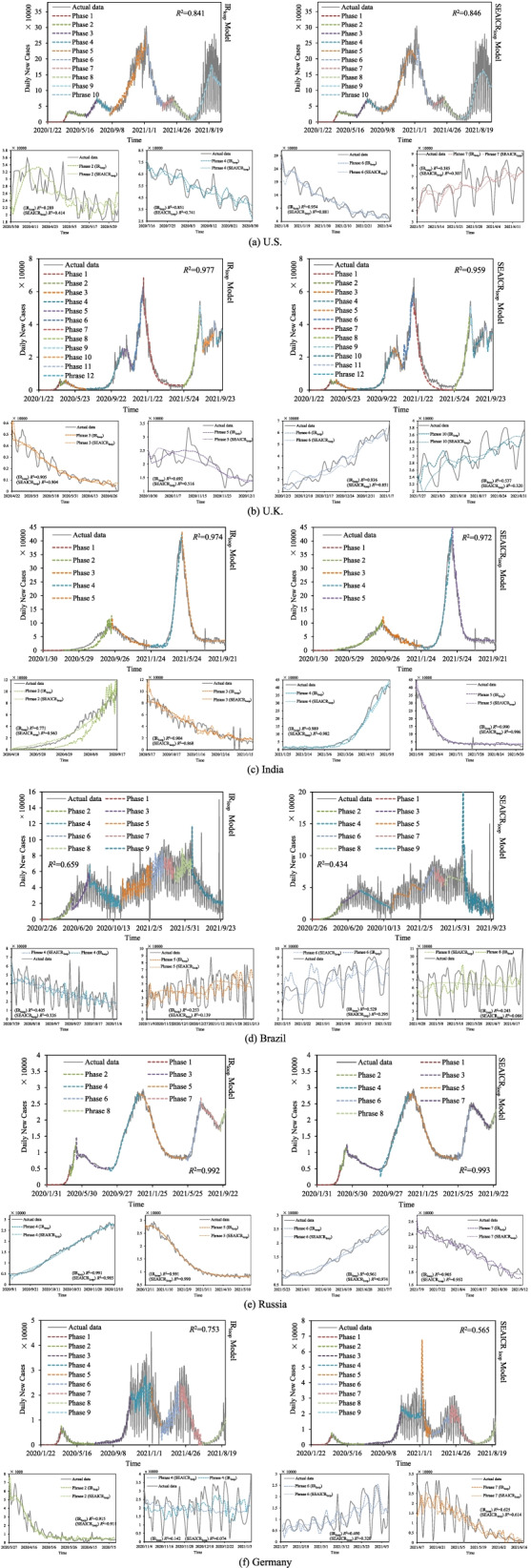
The fitting results of COVID-19 historical data

The parameters of the dynamical model express the epidemic trends under the countries’ state at that time, such as the intensity of interventions, economic development, and population activity. The fitting of historical data has two purposes. One is to obtain the number of different groups as an initial input parameter for the new prediction at the next moment, and the other is to evaluate the model parameters at different stages to select them to predicting future epidemics. The transmission stages of COVID-19 were divided using the methods of curve smooth and first-order difference. In the initial phase, there are no human interventions, so NO_2_ will not be considered (*ε* = 0). The genetic algorithm was used to calculate the optimal parameters with 100 cycles to avoid local optima. The parameters with the best fit-goodness (R^2^) as shown in Fig. 4 and Tables 2 and 3, and the time is from January 22, 2020 to September 30, 2021.

**Table 2:** The estimated parameters of the IR_loop_ model at different stages

Country	Stage	*p*_1_	*p*_2_/ *p*_5_	*p*_3_/ *p*_6_	*p*_4_/ *p*_7_	*ε*_0_	*ε*_1_(×10^−3^)	*γ*	*R*^2^
U.S.	1	0.293	0.138	3.660	94.951	\	\	0.100	0.936
	2	0.245	0.196	0.102	3.588	0.127	0.727	\	0.289
	3	0.425	0.203	0.005	9.569	0.405	1.437	\	0.964
	4	0.217	0.029	0.066	1.493	0.108	0.839	\	0.851
	5	0.334	0.012	0.044	2.914	0.234	0.459	\	0.870
	6	0.934	0.017	0.063	2.079	0.832	0.171	\	0.954
	7	0.002	0.211	0.000	85.210	0.003	0.121	\	0.385
	8	0.001	0.527	0.000	5.426	0.162	0.069	\	0.844
	9	0.405	0.006	0.063	51.481	0.305	0.082	\	0.559
	10	0.531	0.015	0.016	7.067	0.434	0.004	\	0.103
	Prediction by predicted NO_2_	0.047	0.707	0.227	57.699	0.650	0.000	\	0.064
	Prediction by actual NO_2_	0.882	0.005	0.068	1.401	0.781	-0.076	\	0.284
U.K.	1	0.204	0.899	3.592	75.596	\	\	0.032	0.907
	2	0.283	0.834	0.934	89.311	0.187	2.485	\	−3.099
	3	0.143	0.070	0.075	0.157	0.108	0.259	\	0.905
	4	0.390	0.023	0.167	82.711	0.354	0.271	\	0.951
	5	0.142	0.014	0.292	20.555	0.102	0.082	\	0.692
	6	0.055	0.031	0.086	4.131	0.030	0.244	\	0.936
	7	1.033	0.035	0.075	0.000	1.000	0.000	\	0.970
	8	0.240	1.893	0.042	178.847	0.208	0.055	\	0.975
	9	0.054	1.569	0.001	0.794	0.797	0.217	\	0.947
	10	0.221	1.955	1.299	42.713	0.184	0.175	\	0.537
	11	0.612	0.291	0.369	23.030	0.865	0.040	\	0.829
	12	0.589	0.307	0.001	0.464	0.704	0.000	\	0.556
	Prediction by predicted NO_2_	0.566	0.001	0.286	0.000	0.531	−0.017	\	0.224
	Prediction by actual NO_2_	0.885	0.000	1.000	100.000	0.849	-0.058	\	0.334
India	1	0.199	0.002	0.434	52.312	\	\	0.064	0.812
	2	0.872	0.030	1.000	0.000	0.886	−10.077	\	0.771
	3	0.474	0.041	0.042	0.000	0.407	0.082	\	0.904
	4	0.248	0.022	0.143	74.240	0.182	0.174	\	0.989
	5	0.622	0.037	0.083	0.644	0.556	0.000	\	0.990
	Prediction by predicted NO_2_	0.563	0.465	0.128	137.253	0.959	0.503	\	0.620
	Prediction by actual NO_2_	0.325	0.094	0.000	99.734	0.307	-0.007	\	0.855
Brazil	1	0.357	0.967	1.922	53.753	\	\	0.044	0.678
	2	0.466	0.000	0.967	79.430	0.339	−2.163	\	0.735
	3	0.914	0.124	0.958	92.341	0.846	0.642	\	−0.303
	4	0.812	0.049	0.051	0.003	0.764	0.384	\	0.405
	5	0.950	0.017	2.000	0.000	0.916	0.602	\	0.253
	6	0.525	0.018	1.555	103.467	0.493	0.681	\	0.529
	7	0.362	0.060	0.004	6.939	0.343	0.367	\	0.099
	8	0.440	0.759	1.756	59.232	0.393	0.157	\	0.243
	9	0.951	0.005	0.043	0.145	0.906	0.029	\	0.358
	Prediction by predicted NO_2_	0.935	0.005	0.055	0.000	0.890	0.060	\	0.348
	Prediction by actual NO_2_	0.847	0.005	0.031	0.587	0.803	0.033	\	0.510
Russia	1	0.212	0.020	0.289	42.495	\	\	0.086	0.996
	2	0.754	0.000	0.998	52.113	0.611	−4.018	\	0.628
	3	0.110	0.071	0.048	0.000	0.019	−0.018	\	0.884
	4	0.546	0.008	0.111	28.932	0.456	−0.051	\	0.991
	5	0.520	0.012	0.045	26.391	0.432	−0.003	\	0.991
	6	0.689	0.008	0.046	45.131	0.602	0.013	\	0.961
	7	0.092	0.013	0.009	78.782	0.010	0.015	\	0.905
	8	0.829	0.712	0.277	43.082	0.739	0.052	\	0.855
	Prediction by predicted NO_2_	0.889	0.012	0.035	62.029	0.802	0.007	\	−0.449
	Prediction by actual NO_2_	0.572	0.005	0.092	0.276	0.487	-0.070	\	0.823
German	1	0.295	0.663	4.294	73.495	\	\	0.099	0.918
	2	0.111	0.225	0.129	0.000	0.008	0.418	\	0.915
	3	0.290	0.038	0.119	100.000	0.186	0.276	\	0.857
	4	0.999	0.032	0.120	0.176	0.886	1.221	\	0.142
	5	0.450	0.156	0.009	199.726	0.477	0.006	\	0.331
	6	0.858	0.005	0.158	28.293	0.758	0.283	\	0.490
	7	0.210	0.017	0.028	55.876	0.117	0.381	\	0.625
	8	1.044	0.011	0.046	99.842	0.944	0.034	\	0.654
	9	0.509	0.530	0.398	133.925	0.937	0.110	\	0.427
	Prediction by predicted NO_2_	0.476	0.016	0.044	71.132	0.376	0.152	\	0.399
	Prediction by actual NO_2_	0.639	0.253	1.259	190.671	0.788	0.350	\	0.262

The closer *R*^2^ ∈ (−∞, 1] is to 1, the closer the real value is to the predicted value

**Table 3:** The estimated parameters of the SEAICR_loop_ model at different stages

Country	Stage	*p*_1_	*p*_2_/*p*_5_	*p*_3_/*p*_6_	*p*_4_/*p*_7_	*ε*_0_	*ε*_1_	*θ*(×10^−4^)	*φ*	*R*^2^
U.S.	1	0.000	0.001	0.625	53.153	\	\	0.975	0.021	0.979
	2	0.002	0.019	1.000	0.212	0.000	0.019	\	\	0.414
	3	0.004	0.016	0.104	26.084	0.282	0.036	\	\	0.931
	4	0.010	0.019	0.080	2.044	0.504	0.022	\	\	0.746
	5	0.003	0.354	0.043	123.634	0.471	0.005	\	\	0.885
	6	0.327	0.232	1.00	26.383	0.542	0.013	\	\	0.881
	7	0.009	0.817	0.073	103.328	0.418	0.027	\	\	0.307
	8	0.003	0.033	0.069	0.648	0.899	0.017	\	\	0.821
	9	0.001	0.018	0.117	40.241	0.718	0.000	\	\	0.576
	10	0.013	0.004	1.000	15.132	0.662	0.000	\	\	0.087
U.K.	1	0.000	0.021	0.123	68.784	\	\	0.002	0.010	0.974
	2	0.006	0.738	0.760	128.029	0.385	−0.039	\	\	−0.370
	3	0.001	0.012	0.078	0.000	0.371	−0.023	\	\	0.904
	4	0.003	0.488	0.089	113.493	0.988	−0.010	\	\	0.955
	5	0.288	0.167	1.000	20.777	0.910	0.000	\	\	0.516
	6	0.028	1.000	0.226	30.958	0.228	−0.013	\	\	0.851
	7	0.822	0.004	0.232	100.000	0.221	0.000	\	\	0.922
	8	0.002	0.755	0.078	81.526	0.260	−0.009	\	\	0.967
	9	0.026	0.725	1.000	0.000	0.544	−0.021	\	\	0.948
	10	0.010	0.996	0.036	161.878	0.281	0.012	\	\	0.320
	11	0.007	0.080	1.000	0.002	0.290	0.012	\	\	0.748
	12	0.006	0.791	0.312	36.193	0.155	0.004	\	\	0.398
India	1	0.000	0.322	0.127	150.974	\	\	0.998	0.056	0.812
	2	0.000	0.014	0.024	176.259	0.894	0.089	\	\	0.963
	3	0.001	0.006	0.041	0.068	1.000	0.060	\	\	0.868
	4	0.000	0.033	0.068	95.547	1.000	0.019	\	\	0.982
	5	0.002	0.012	0.141	15.817	1.000	0.036	\	\	0.986
Brazil	1	0.000	0.283	0.169	71.120	\	\	0.773	0.015	0.778
	2	0.000	0.042	0.069	63.196	1.000	0.005	\	\	0.863
	3	0.003	0.306	0.019	137.405	1.000	0.004	\	\	0.192
	4	0.010	0.062	0.021	58.595	1.000	−0.009	\	\	0.326
	5	0.026	0.022	1.000	53.233	0.696	−0.005	\	\	0.139
	6	0.000	0.593	0.021	152.796	0.745	0.022	\	\	0.295
	7	0.041	0.003	1.000	20.555	0.905	−0.050	\	\	0.129
	8	0.023	1.000	1.000	56.585	0.963	−0.001	\	\	0.066
	9	0.010	0.006	0.202	50.338	1.000	− 0.092	\	\	−1.057
Russia	1	0.000	0.019	0.140	92.709	\	\	0.275	0.016	0.996
	2	0.002	1.000	0.076	94.411	0.339	−0.020	\	\	0.923
	3	0.002	0.006	0.024	13.978	0.433	0.004	\	\	0.962
	4	0.000	0.068	0.031	84.262	0.901	−0.002	\	\	0.985
	5	0.017	0.030	0.076	46.429	0.994	0.000	\	\	0.990
	6	0.002	0.020	0.099	19.842	0.695	0.003	\	\	0.974
	7	0.017	0.004	0.076	24.714	0.715	0.003	\	\	0.952
	8	0.007	0.960	0.199	41.889	0.531	0.015	\	\	0.813
German	1	0.000	0.018	0.146	67.301	\	\	0.001	0.018	0.941
	2	0.000	0.010	0.132	0.941	0.401	0.000	\	\	0.911
	3	0.002	0.891	0.087	143.050	1.000	0.019	\	\	0.857
	4	0.043	1.000	1.000	63.241	1.000	-0.024	\	\	0.074
	5	0.000	0.023	1.000	29.297	1.000	-0.082	\	\	−2.346
	6	0.011	0.016	0.402	29.240	0.998	0.015	\	\	0.320
	7	0.002	0.035	0.137	22.234	0.863	−0.085	\	\	0.614
	8	0.000	1.000	0.057	151.477	1.000	−0.108	\	\	0.705
	9	0.001	0.008	1.000	50.425	1.000	0.025	\	\	-0.149

Figure 4 shows: the IR_loop_ and the SEAICR_loop_ models can achieve the epidemic prediction, which is reflected in the fitting characteristics with large fluctuations and multiple peaks. Among them, the IR_loop_ model can achieve better results overall than the SEAIR_loop_ model, especially for epidemic curves with large fluctuations. This is mainly because the parameters of the IR_loop_ model have better actual validation and the SEAIR_loop_ model contains a large number of parameters that cannot be validated (the actual data contains only the number of confirmed people and recovered people and dead people), which also makes the model more ambiguous and uncertain. However, in the second phase in India and the US, the SEAIR_loop_ model achieved better results. This is mainly because the number of infected cases is small at the beginning of COVID-19 and the ambiguity and uncertainty of the model are relatively small. The SEAIR_loop_ model has a more complete dynamical mechanism, which can better describe the transmission process of COVID-19. However, this ambiguity and uncertainty will continue to be superimposed as the epidemic continues, eventually leading to the prediction of the SEAIR_loop_ model does not achieve better results. In addition, the SEAIR_loop_ model can obtain better prediction results mostly for curves with smooth and small volatility, which also reflects that the IR_loop_ model has an advantage in terms of volatility.

### Time-series predictions of NO_2_ concentrations

**Fig. 5:**
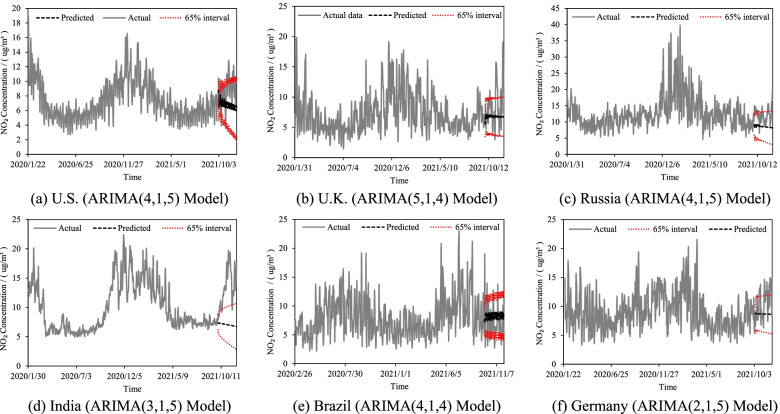
Prediction results of NO_2_ concentrations (the red dot is the upper and lower interval value of 65% of the predicted value)

To predict the development of COVID-19 in the future, the intensity of human interventions is a key factor. NO_2_ concentrations can reflect indirectly the intensity of interventions. The changes of NO_2_ have stability in a short period and have the timing characteristics of seasonality, long-term trends, stochastic fluctuations, and cyclic changes in time series. Therefore, the ARIMA model was used to predict the NO_2_ concentrations from October 1, 2021 to November 30, 2021, which provides a data basis for the later epidemic prediction in the future. The results are shown in Fig. 5.

As can be seen in Fig. 5, the predicted NO_2_ concentrations remain relatively stable, and the main trends maintain the upward or downward of the previous phase. This is mainly because NO_2_ concentrations are meteorological factors that vary continuously over time and space. It is difficult to have large changes aggregated nationwide in short-term time. The predictions of the ARIMA model are less volatile compared to the actual NO_2_ concentrations because multiple predictions are averaged. This treatment ensures the stability of the NO_2_ concentrations trends. Among them, the prediction results for India differed from the actual one, mainly due to the sudden relaxation of the policy that led to a sharp increase in NO_2_. However, such sudden events are difficult to predict.

### Prediction of the development trends of COVID-19 in the future

The epidemic prediction is of great importance for epidemic prevention and control. The trends of the infection curve are not the same due to the unknown nature of the future and the variability of viruses. Meanwhile, sample data for the last phase of the epidemic curves are usually too small, so it is unreasonable to estimate the model parameters for that phase using only these data. How to determine the model parameters for future curves will be a key issue for epidemic prediction.

**Fig. 6:**
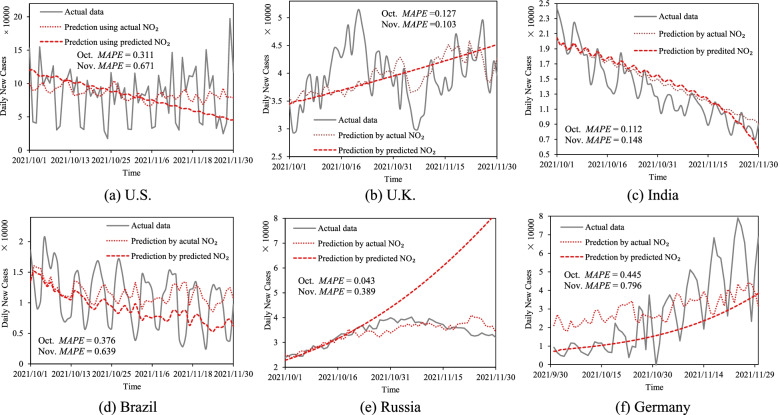
The prediction of COVID-19 (The numbers are the accuracy of the prediction by predicted NO_2_)

The parameters of dynamics models can control the rise or fall of the overall trend of the epidemic curve. However, the changes in the overall trend of the epidemics are due to the occurrence of unpredictable events, such as secondary outbreaks caused by virus mutations and rapid declines in the epidemic caused by the intensification of prevention and control measures. Thus, the prediction of the epidemic inflection point is very difficult. The significance of prediction is to let people know how the epidemic will develop in the future by continuing the current epidemic status, national interventions, and economic status. Then, this can provide decision support information to regulate the current epidemic prevention and control status in response to future changes. The parameters of historical epidemic curves contain the development trends of COVID-19 under the influence of the current epidemic condition, human intervention status, and national economic condition. Therefore, the approximate reflection of the future epidemic trends can be expressed by these parameters. The model parameters with the same trends in the historical stages were selected to predict future epidemic development. Meanwhile, the small amount of sample data in the last stage could be used as validation to select the results. Results were shown in Fig. 6 and Table 1 (time from October 1, 2021, to November 30, 2021). Mean Absolute Percentage Error ($MAPE=\frac{1}{n}\sum \limits_{i=1}^n\mid \left({y}_i-{\hat{y}}_i\right)\mid$) was used to measure the accuracy of results. As the effects of temperature and humidity on COVID-19 are still controversial, the influence of this factor on the results is excluded from the experiments [22].

As can be seen from Fig. 6, the prediction results have good stability in the overall trend. The prediction accuracy (*MAPE*) of the US, UK, India, Brazil, Russia, and Germany in October is 0.331, 0.127, 0.112, 0.376, 0.043, and 0.445, respectively. There are two main problems: firstly, the local fluctuations of the predicted results are low, mainly because the NO_2_ concentrations used were the mean of ARIMA model prediction results, and they were relatively stable. This treatment can better ensure that the general trend of NO_2_ concentrations change is correct because the long-term prediction of future NO_2_ concentrations is difficult to achieve. Secondly, in Russia, the United Kingdom, and the United States, the predicted trends exit opposite to the actual situation. After introducing the actual NO_2_ concentrations, the prediction curve has a great improvement, but the overall trend remains unchanged, which is verified obviously in the UK. This is mainly because NO_2_ concentrations are more the adjustment parameter of curve volatility, and the parameters of the dynamical model are the main factor controlling the overall trend change. However, these changes in overall trend in epidemic trends are due to abrupt and unpredictable events, for example, omicron infection was found in the United States and the United Kingdom in late November 2021. The significance of epidemic prediction is to let people know how the epidemic will develop in the future by continuing the current epidemic status, national prevention, and control, and economic status. Therefore, the prediction model in this study pays more attention to the epidemic situation under the continuation of the current prevention and control status.

## Discussion

COVID-19 is still in a very serious state and is also showing multiple different-scale outbreaks. All countries need to prepare for proactive prevention and control rather than passive defense. The prediction of the epidemic is important assistant information. This study proposed a novel model to predict the COVID-19 pandemic with the characteristics of long-time series, multiple peaks, and large fluctuations.

There are several points worth explaining here. Firstly, the problem of long-time series refers to the historical duration of the epidemic, such as foreign epidemics that have lasted more than 2 years. Our model can well describe the transmission process of such a long-time series of infectious diseases. The prediction of long-time series refers to the forecasting for almost 1–3 months under the continuation of the current state of prevention and control, which is also more suitable for the asymmetric and non-smooth epidemic curves with large fluctuations. Secondly, the fitting of historical epidemics is also very good using our model. However, there are still two limitations to the prediction of future epidemics. First, the model is difficult to predict the outbreak of new epidemics and the inflection point of epidemics. The introduction of NO_2_ concentrations is only a parameter to regulate the fluctuation feature of the epidemic curve and the parameters of the dynamic models of infectious diseases are a major factor in regulating the upward or downward trend of the epidemic curve. However, the changes in the overall trend of the epidemics are due to the occurrence of unpredictable events, such as secondary outbreaks caused by virus mutations and rapid declines caused by the intervention of prevention and control measures. Authors think that the significance of epidemic prediction should be to let people know how the epidemic will develop in the future by continuing the current epidemic status, national prevention, and control, and economic status. Thus, the future development of COVID-19 can be predicted by selecting epidemic parameters with similar trends in past periods. Because these historical parameters contain the development trends of COVID-19 under the current conditions of epidemic conditions, human prevention and control, and the national economy. This study provides an idea to predict the trend of future epidemics if the current prevention and control situation continues. This prediction is not focused on the specific daily changes in the number of infected cases and more reflects the long-term trends of the future epidemics. Second, there is still room to improve the volatility of the future prediction curve. This fluctuation is mainly caused by the restriction on people’s activities of human intervention measures, which is reflected by NO_2_ concentrations. When we bring in the real NO_2_ data, the prediction curve has better improved. But it is difficult to predict well the NO_2_ concentrations for a longer period in the future. The predictions of the ARIMA model are very different in the multiple simulations. Therefore, we choose the mean value of multiple simulations so that prediction curves are relatively smooth. This way can avoid subjective errors but also lead to the unobvious fluctuations. Finally, the epidemiological mechanisms of the SEAICR_loop_ model are more complex. The more complex mechanism also leads to the fact that a large number of parameters cannot be verified by actual report data, which will increase the fuzziness and uncertainty of the model. Therefore, the prediction result of the SEAICR_loop_ model is better than that of the IR_loop_ model only in the early stage of the epidemic because the number of infected people is relatively small in this term. However, as the number of infected people increases, the fuzziness of the model will increase, and the advantages of the IR_loop_ model will be more reflected.

## Conclusion

This study proposed a new dynamical model of infectious diseases, which aims to quantify the COVID-19 pandemic with the characteristics of long-time series, multiple peaks, and large fluctuations and predict the development trend of the epidemic. Through experimental results, the model could realize the epidemic prediction with high accuracy and reasonableness, especially for the epidemic curve with a large fluctuation. The method also breaks the limitation of the traditional epidemic curve with smooth and symmetrical characteristics. The goodness-of-fit *R*^2^ of the prediction for the US, UK, India, Brazil, Russia, and Germany were 0.841, 0.977, 0.974, 0.659, 0.992, and 0.753, respectively. The model used the trends of epidemic changes in the historical stages as the empirical parameters and the predictions have high consistency in the overall trend. The prediction accuracy (*MAPE*) of the US, UK, India, Brazil, Russia, and Germany in October is 0.331, 0.127, 0.112, 0.376, 0.043, and 0.445, respectively.

The model is still in an early stage of research and still lacks the incorporation of a large amount of data, such as local medical conditions, the degree of population aging, and the travel characteristics of the population, which would greatly reduce the uncertainty of the epidemic prediction. The strength of the model lies more in describing the characteristics of long series, multiple peaks, and large fluctuations for COVID-19, which will also help to understand and mitigate the impact of the epidemic and provide a valuable reference for decision-makers.

## Supplementary Information

**Additional file 1.**

## Data Availability

The datasets generated during the current study are available in the zenodo repository, [10.5281/zenodo.6220224]. The code and original data have been uploaded to GitHub, [https://github.com/daihaoran1/Global-Prediction-Model-for-COVID-19-Pandemic-with-the-Characteristcs-of-the-Multiple-Peaks-and-Loca].
